# Metabolic dysfunction associates with neuroimaging measures of brain structure and connectivity

**DOI:** 10.3389/fnins.2026.1889255

**Published:** 2026-09-25

**Authors:** Zachary D. Green, Sid E. O’Bryant, Leigh A. Johnson, Jeffrey M. Burns, Eric D. Vidoni, Jill K. Morris

**Affiliations:** 1 University of Kansas Alzheimer’s Disease Research Center, Kansas City, KS, United States; 2 Department of Neurology, University of Kansas School of Medicine, Kansas City, KS, United States; 3 University of North Texas Health Science Center, Fort Worth, TX, United States

**Keywords:** brain, connectivity, glucose, HbA1c, hyperglycemia, structure

## Abstract

**Introduction:**

Metabolic syndrome (MetS) and insulin resistance are established risk factors for Alzheimer’s disease (AD) and have been linked to alterations in brain structure and function, including within the Default Mode Network (DMN).

**Methods:**

Here, we examined associations between metabolic dysfunction, brain structure, and DMN functional connectivity using data from the Health and Aging Brain Study: Health Disparities (HABS-HD) cohort. Participants were classified as being cognitively unimpaired (CU; *n* = 1,045; mean age = 66.1 years; 65% female), or having mild cognitive impairment (MCI; *n* = 186; mean age = 66.5 years; 52% female) or dementia (*n* = 76; mean age = 69.0 years; 47% female).

**Results:**

Across diagnostic groups, cortical thickness in AD-vulnerable regions varied by both cognitive status and HbA1c grouping, with the strongest effects observed in the CU cohort. HbA1c grouping was also associated with white matter microstructure, including stepwise reduc tions in fractional anisotropy of the anterior corona radiata across glycemic cat egories (normoglycemia > prediabetes > diabetes) in CU individuals. Functional connectivity of the posterior DMN (pDMN) showed main effects of both cognitive status and HbA1c grouping. Using a continuous index of cardiometabolic risk (MetS-Z), pDMN connectivity decreased linearly with increasing cardiometabolic burden in the CU cohort, whereas other DMN subnetworks exhibited positive associations with MetS-Z.

**Discussion:**

These findings suggest network-specific associations between cardiometabolic risk and DMN organization that are detectable in indi viduals with MCI, a clinical state preceding dementia, as well as in some degree within cognitively unimpaired individuals. Longitudinal analyses are needed to determine whether metabolic dysfunction predicts divergent trajectories of DMN connectivity over time.

## 1 Introduction

Alzheimer’s disease (AD) is the most common cause of dementia, affecting over 6.2 million Americans (Alzheimer's Association Report, 2023). Although AD is neuropathologically defined by the presence of amyloid beta (Aβ) and tau neurofibrillary tangles, it is a multifactorial disease also associated with metabolic impairment, vascular dysfunction, and neuroinflammation (Sweeney et al., 2019). Type 2 diabetes and insulin resistance are well-established risk factors for AD, (Cheng et al., 2011, 2012; Luchsinger et al., 2007) and both elevated fasting glucose levels and a high glycemic diet associate with Aβ prior to clinical symptoms (Li et al., 2016; Taylor et al., 2017). Type 2 diabetes and metabolic syndrome (MetS), which includes vascular risk factors, may contribute to cognitive decline through altering glycemic regulation, hypoglycemia, systemic inflammation, or by contributing to psychiatric disease (anxiety, depression) (Feinkohl et al., 2015). Type 2 diabetes, a clinical disorder characterized by chronic dysregulation of glucose metabolism, is associated with accelerated cognitive decline (Morris et al., 2014), reduced global brain volume, accelerated hippocampal volume loss (Burns et al., 2007), and increased Aβ deposition (Morris et al., 2016). These dysmetabolic conditions are thought to contribute to vascular dysfunction and systemic inflammation, further potentiating risk for AD (Morris et al., 2014).

Several brain imaging approaches offer insight to the relationship of metabolism and brain health. For example, blood oxygen level dependent imaging at rest (resting-state fMRI) allows characterization of regional co-activation patterns, often called functional connectivity. The regions demonstrating high degrees of connectivity in the resting state include posterior cingulate cortex, inferior parietal cortex, midline/lateral frontal cortex, and the medial temporal cortex, together called the Default Mode Network (DMN). Disruptions in DMN functional connectivity have been observed in cognitively unimpaired individuals with type 2 diabetes, with prediabetic individuals exhibiting an intermediate connectivity phenotype between normoglycemic controls and diabetic participants (Macpherson et al., 2017). In separate cohorts, functional connectivity in type 2 diabetes participants was also decreased in the DMN, with more pronounced disruptions highlighted in individuals with co-occurring mild cognitive impairment (MCI) (Yang et al., 2016).

DMN functional connectivity is separately a descriptive phenotype of AD. The DMN consists of highly metabolic brain regions that also demonstrate marked glucose hypometabolism in AD (Mosconi et al., 2009), MCI (He et al., 2015), and even in cognitively unimpaired individuals with genetic risk for AD (Jagust et al., 2012). While cardiometabolic risk factors do not affect only the DMN, these converging observations highlight the DMN as a specific metabolically vulnerable network at the intersection of cardiometabolic dysfunction and neurodegeneration. This is particularly relevant in AD, as DMN regions are amongst the first to accumulate AD neuropathology (Palmqvist et al., 2017). Moreover, existing studies examining DMN connectivity in the context of metabolic disease or dementia have largely relied on modest sample sizes and demographically homogeneous cohorts, limiting generalizability and the ability to disentangle early metabolic effects from downstream neurodegenerative processes.

In the present work, we investigated functional connectivity changes in cognitively unimpaired and cognitively impaired older adults to evaluate relationships with cardiometabolic risk (defined cardiometabolic risk using fasting hyperglycemia in conjunction with the MetS-Z continuous scale of metabolic syndrome severity). While MetS-Z does not classify participants into specific clinically validated groups based on metabolic syndrome severity, this continuous index reflects a broader constellation of metabolic syndrome components than HbA1c alone. Using multimodal neuroimaging, we examined cortical thickness, white matter microstructure, and functional connectivity of the DMN and its subnetworks in cognitively unimpaired individuals, participants with MCI, and individuals with AD. The rationale for a multimodal analysis allows for a more comprehensive characterization regarding the degree to which metabolic function is associated with brain health. We hypothesized that metabolic dysfunction would be associated with alterations in DMN functional connectivity, with detectable effects emerging even in groups with cognitive impairment not yet meeting the clinical definition of dementia (i.e., MCI). By integrating categorical and continuous measures of metabolic risk within a well-characterized and diverse sample, this work aims to clarify early neural correlates of cardiometabolic dysfunction relevant to AD risk.

## 2 Materials and methods

We leveraged data collected through the non-Hispanic White (NHW) & Mexican-American (Hispanic) arms of the Health & Aging Brain Study–Health Disparities (HABS-HD) study, which aims to identify health disparities of aging and AD. Detailed information about the HABS-HD study protocol has been published (O'Bryant et al., 2021). De-identified data from the HABS-HD study used in this study is publicly available on request (https://apps.unthsc.edu/itr/researchers).

### 2.1 Participants

Participants in this analysis were community-dwelling adults aged 50 or older and all underwent cognitive evaluation via consensus review. Mild cognitive impairment was defined as (1) self or other reported cognitive change, (2) CDR sum-of-boxes of 0.5 to 2.0, and (3) performance at or below 1 standard deviations below z-score adjusted norms on one or more cognitive tests in the HABS-HD battery. Dementia status was defined by the same criteria, but with a CDR sum-of-boxes equal to or greater than 2.5, and performance at or below 2 standard deviations below z-score adjusted norms on two or more cognitive tests. Notably, this dementia classification was informed clinically, and not with the use of pathology or biomarkers. We included all participant datasets with structural or functional MRI scans that passed visual inspection and an automated processing pipeline (Figure 1).

**Figure 1:**
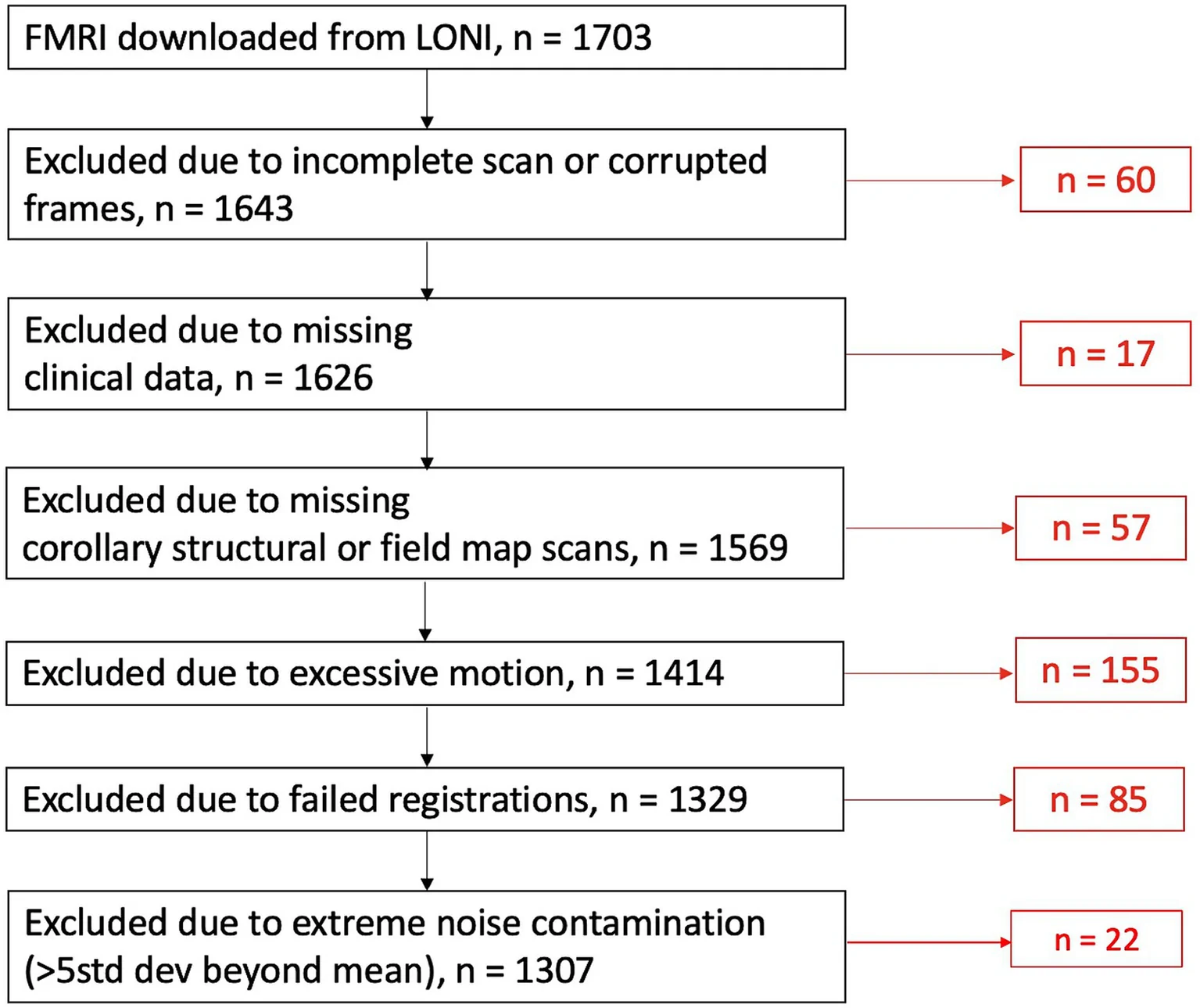
Sample size determination of available data in the HABS-HD. Data was downloaded from the Laboratory of Neuro Imaging (LONI) Image and Data Archive. Images were excluded (shown in red) at baseline based on incomplete data, having missing data in the clinical dataset, missing or incomplete field maps acquisition. During pre-processing, individuals whose functional data failed registration or were flagged by FSL for excessive motion were also excluded. Finally, as the FSL threshold for flagging motion is rather conservative, we also excluded individuals who had absolute displacement >5 standard deviations of the rest of the sample.

### 2.2 Metabolic measures and MetS-Z score

Hemoglobin A1c (HbA1c) measures glycated hemoglobin over the preceding ~3 months and was used to define pre-diabetes and diabetes groupings according to American Diabetes Association criteria. Normal glycemia was considered to be 5.6 and lower, prediabetes from 5.7 to 6.4 (inclusive), and diabetes 6.5 and higher. We chose this measure due to its clinically interpretable classification. To examine broader systemic metabolic risk, rather than a diagnosis of diabetes or MetS alone, we also utilized the MetS Z-score. The MetS Z-Score is a continuous measure of metabolic risk (Gurka et al., 2014), integrating the five components of MetS diagnostic criteria: systolic blood pressure (sBP), high density lipoprotein (HDL), triglycerides, fasting glucose, and obesity (abdominal circumference or BMI) (Grundy et al., 2005). Thus, MetS-Z can be interpreted to reflect broader systemic metabolic burden. MetS-Z was not used to assign participants to discrete groups.

### 2.3 Neuroimaging acquisitions and structural data

The MRI acquisition protocol for this study is similar to Alzheimer’s Disease Neuroimaging Initiative (ADNI) 3, with specific sequence parameters used in HABS-HD available in O'Bryant et al. (2021). The MRI scanner used for all scans in this current work was a 3 T Siemens MAGNETOM Skyra. The scans used for all participants in this current study were the T1-weighted whole brain volumetric spoiled Magnetization-Prepared Rapid Gradient (MPRAGE; 1.1 × 1.1 × 1.2 mm^3^; TR = 2,300 ms; TE = 2.93 ms, TI = 900 ms), Diffusion Tensor Imaging (DTI; 1.7 × 1.7×2.5 mm^3^; TR = 4,000 ms; TE = 92 ms; 64 direction, b = 1,000 s/mm^2^) and BOLD resting-state functional MRI (rsfMRI; 3.3 × 3.3 × 3.0 mm; TR = 3,380 ms; TE = 30.0 ms; 90 deg. flip angle, 150 volumes, eyes open) with associated field mappings. We independently processed the resting-state fMRI was for the present study. Both MPRAGE (cortical thickness, hippocampal volume, Freesurfer 5.3.0’s *recon-all*) and DTI summary measures (fractional anisotropy, FA; axial diffusivity, AxD; mean diffusivity, MD; radial diffusivity, RD (Hall et al., 2021) of regions-of-interest were provided by the HABS-HD study team.

We limited our analyses to DMN-related regions and regions key to early AD pathophysiologic change to limit the degree of multiple comparisons correction (Sidak) required to control Type I error. This included the precuneus, superior parietal lobule, inferior parietal lobule, insula, supramarginal gyrus, bank of the superior temporal sulcus, entorhinal cortex, and the hippocampus. We also specifically examined an AD “Meta-ROI” measure of cortical thickness, (defined as a surface-area weighted average of entorhinal, inferior temporal, middle temporal, and fusiform gyri bilateral thicknesses) in addition to individual regional measures to capture a broader, disease relevant pattern of neurodegeneration rather than relying solely on changes within anatomical regions. The AD Meta ROI, as defined by Jack et al. (2019) integrates regions previously identified as sensitive to AD related neurodegeneration and provides a summary measure of cortical vulnerability across these areas. This approach reduces the influence of region-specific variability and allows us to evaluate whether cardiometabolic risk is associated with a more generalized pattern of AD-relevant neurodegeneration. White matter tracts examined through DTI included the corticospinal tract, limbs of the internal capsule (anterior; posterior), thalamic radiations (posterior), corona radiata (anterior; superior; posterior), cingulate (cingulate; hippocampal), longitudinal fasciculus (superior), fronto-occipital fasciculus (superior; inferior), uncinate fasciculus, and the corpus callosum (genu; body; splenium). All ROIs for both the structural and DTI measures were examined with left and right regions considered separately.

### 2.4 Pre-processing and generation of functional maps

Our processing stream is depicted in Figure 2. All rsfMRI preprocessing steps and analyses were performed using FMRIB’s Software Library (FSL) version 6.0. The rsfMRI data underwent standard processing, including FSL’s MCFLIRT motion correction tool (Jenkinson et al., 2002) (https://fsl.fmrib.ox.ac.uk/fsl/docs/registration/mcflirt.html). This tool realigns the brain volumes to a reference image using rigid body registration to reduce artifacts. In addition, we performed distortion correction utilizing available field maps, BET brain extraction, spatial smoothing using a 5 mm full-width at half-maximum Gaussian kernel, and high pass temporal filtering (100 s). Functional images were registered to standard space utilizing FSL’s FNIRT tool, which first registers the functional data to an individual’s brain-extracted T1-weighted image, and then the MNI152 2 mm atlas.

**Figure 2:**
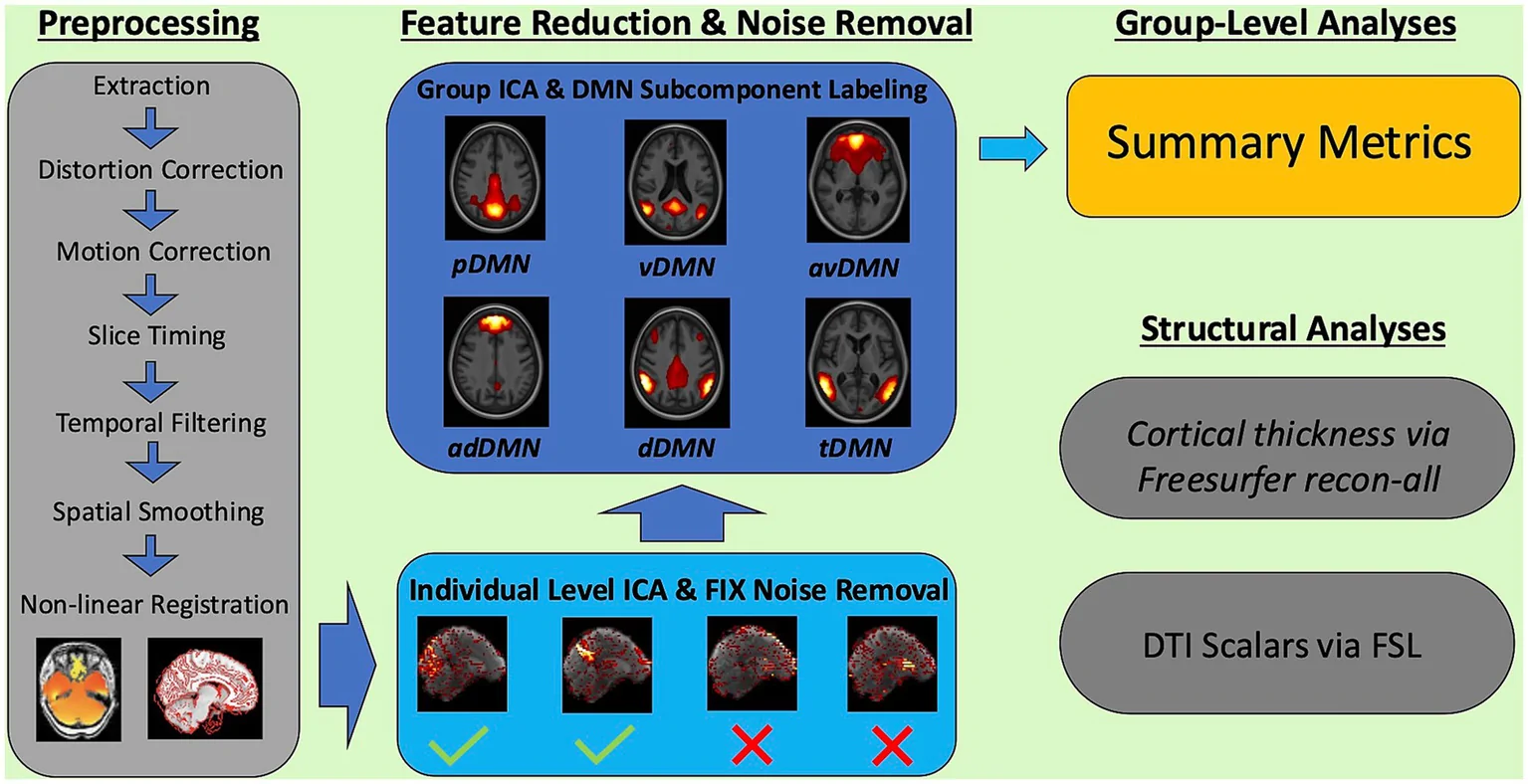
Processing stream for neuroimaging data. Depiction of the flow for pre-processing, processing, and group-level analysis of the functional data, as well as the additional structural analyses. Cortical thickness and DTI scalar data was already processed by the HABS-HD team, and made available through the data request portal.

We utilized a data-driven approach to functional connectivity, namely spatial Independent Component Analysis (ICA) via the FSL Multivariate Exploratory Linear Optimized Decomposition into Independent Components (MELODIC) 3.0. This toolset utilizes ICA to decompose 4D fMRI data sets into their respective spatial and temporal components (Jenkinson et al., 2012), modeling voxel-wise signal as a linear mixture of signals that correspond to underlying structures or “networks,” identified as non-Gaussian components which are statistically independent. MELODIC was used at the single-subject level to separate out both noise and signal-related contributions to the overall signal. We further used FSL’s ICA-based Xnoiseifier (FIX), which functions to automatically classify components extracted at the single-subject level into either signal or noise categories (Griffanti et al., 2014; Salimi-Khorshidi et al., 2014). FIX labeled and removed noise components to maximize the fidelity of our single subject data.

### 2.5 Group-level MELODIC and within-network functional connectivity

At the group level, we utilized MELODIC’s preferred temporal concatenation approach to identify common spatial patterns in the absence of a consistent temporal response (Erhardt et al., 2011). This method reliably identifies AD-relevant resting state networks, such as the default mode network and attentional networks. We verified our extracted group-level ICA components against the MCALT functional atlas to ensure we accurately identified the DMN and relevant subnetworks (Jones et al., 2012).

To generate individual level maps of resting state networks, we used FSL’s Dual Regression tool (Nickerson et al., 2017). First, for each subject, the group-average set of spatial maps is regressed (as spatial regressors in a multiple regression) into the subject's 4D space-time dataset. Next, those timeseries are regressed into the same 4D dataset, resulting in a set of subject-specific spatial maps, one per group-level component.

Finally, we tested for group differences and covariate-parameter estimates in subject-specific spatial maps by using a summary metric approach by taking the median value in each subject-specific, network-specific spatial map found within a thresholded (threshold = 7) and binarized mask of each group-level component of interest.

### 2.6 Statistical analyses

Inference testing outside of imaging space were performed in SPSS v.29. General linear models were used for examining the effect of cognitive status and HbA1c groupings/MetS-Z on brain markers (dependent variables – structural measures, model 1; DTI scalars, model 2, DMN functional connectivity measures, models 3 and 4), and in all cases included sex, age, intracranial volume, and motion (for fMRI analyses) as covariates. Multiple comparisons correction (Sidak) was performed for each univariate post-hoc ANCOVA performed after identification of significance in an overall MANCOVA.

## 3 Results

Table 1 contains demographic, metabolic characterization, and cognitive summary statistics of the 1307 datasets surviving imaging analysis.

**Table 1:** Demographics, metabolic characterization, and cognition of available cohort.

Outcome measure	Total (*N* = 1,307)	CH (*N* = 1,045)	MCI (*N* = 186)	Dementia (*N* = 76)
Age	66.3 (8.7)	66.1 (8.5)	66.5 (9.3)	69 (8.7)
Sex (% Female)	62%	65%	52%	47%
Ethnicity (Mexican American)	53%	50%	64%	56%
BMI	29.8 (5.9)	29.8 (5.9)	30.3 (5.9)	29.0 (6.3)
MetS *Z*-score	0.51 (0.03)	0.48 (0.03)	0.67 (0.07)	0.60 (0.01)
Waist circumference	39.8 (5.7)	39.6 (5.7)	40.6 (5.5)	40.3 (6.4)
sBP	137.5 (20.8)	137.2 (20.6)	139.3 (22.5)	138.2 (20.5)
HDL	53.8 (16.0)	54.7 (16.3)	50.1 (14.0)	51.4 (14.1)
Triglycerides	137.3 (86.6)	135.2 (89.5)	149.2 (74.7)	137.3 (73.0)
Fasting Glucose	109.2 (36.5)	108.3 (35.4)	113.2 (41.1)	112.0 (38.9)
HbA1c	6.0 (1.3)	6.0 (1.3)	6.1 (1.4)	6.1 (1.5)
SCC	41%	37%	55%	61%
MMSE	27.3 (3.2)	28.0 (2.4)	26.1 (3.2)	22.1 (5.8)

Characterization data for the portion of the HABS-HD cohort with intact functional imaging data. Means (standard deviation) and proportions are shown for the entire cohort, as well as for each individual cognitive diagnosis group. CH, cognitively healthy; MCI, mild cognitive impairment. MetS, metabolic syndrome; BMI, body mass index; sBP, systolic blood pressure; HDL, high density lipopolysaccharide; HbA1c, glycated hemoglobin; SCC, subjective cognitive complaint (self-reported); MMSE, mini-mental status exam.

### 3.1 Cortical thickness varies by cognitive status and HbA1c grouping

Cortical thickness comparisons revealed several regions of decreased cortical thickness across the cognitive impairment groups. The extensive list of univariate tests is shown in Supplementary Table 1. Regions that showed an effect of Cognition on cortical thickness included bilateral bank of the temporal sulcus, bilateral entorhinal cortex, bilateral inferior parietal lobes, bilateral precuneus, bilateral superior parietal lobes, bilateral superior temporal lobes, bilateral supramarginal cortex, right insula, and the cortical thickness meta-ROI. Bilateral hippocampal volumes were also evaluated.

Across all significant regions, Sidak-corrected pairwise comparisons demonstrated reduced cortical thickness (or reduced volume in the case of hippocampus) in dementia relative to cognitively unimpaired (CU) individuals. Hippocampal volumes and nearly all regions’ cortical thicknesses demonstrated a difference between MCI and dementia groups, with the exception of the right superior parietal and right superior temporal cortices. Only the right insula (*p* = 0.004) and the cortical thickness meta-ROI (*p* = 0.049) showed significant differences between CU and MCI groups once Sidak-corrected. Hippocampal volumes did not show a difference between CU and MCI groups once Sidak-corrected (left, *p* = 0.063; right, *p* = 0.058).

Regions that were associated with both Cognition and HbA1c grouping included bilateral bank of the temporal sulcus, bilateral inferior parietal lobes, bilateral precuneus, bilateral superior parietal lobes, bilateral temporal lobes, bilateral supramarginal cortex, right insula, and cortical thickness meta-ROI. Bilateral hippocampal volumes (left, *p* = 0.383; right, *p* = 0.747) and bilateral entorhinal cortex thickness (left, *p* = 0.662; right, *p* = 0.878) did not show an effect of HbA1c grouping. All regions which were tested significant for effects of both Cognition and HbA1c grouping were visualized for covariate-adjusted (age, sex, and intracranial volume) cortical thickness in Figure 3. Sidak-adjusted multiple comparisons between HbA1c groupings *within each diagnostic* category are shown.

**Figure 3:**
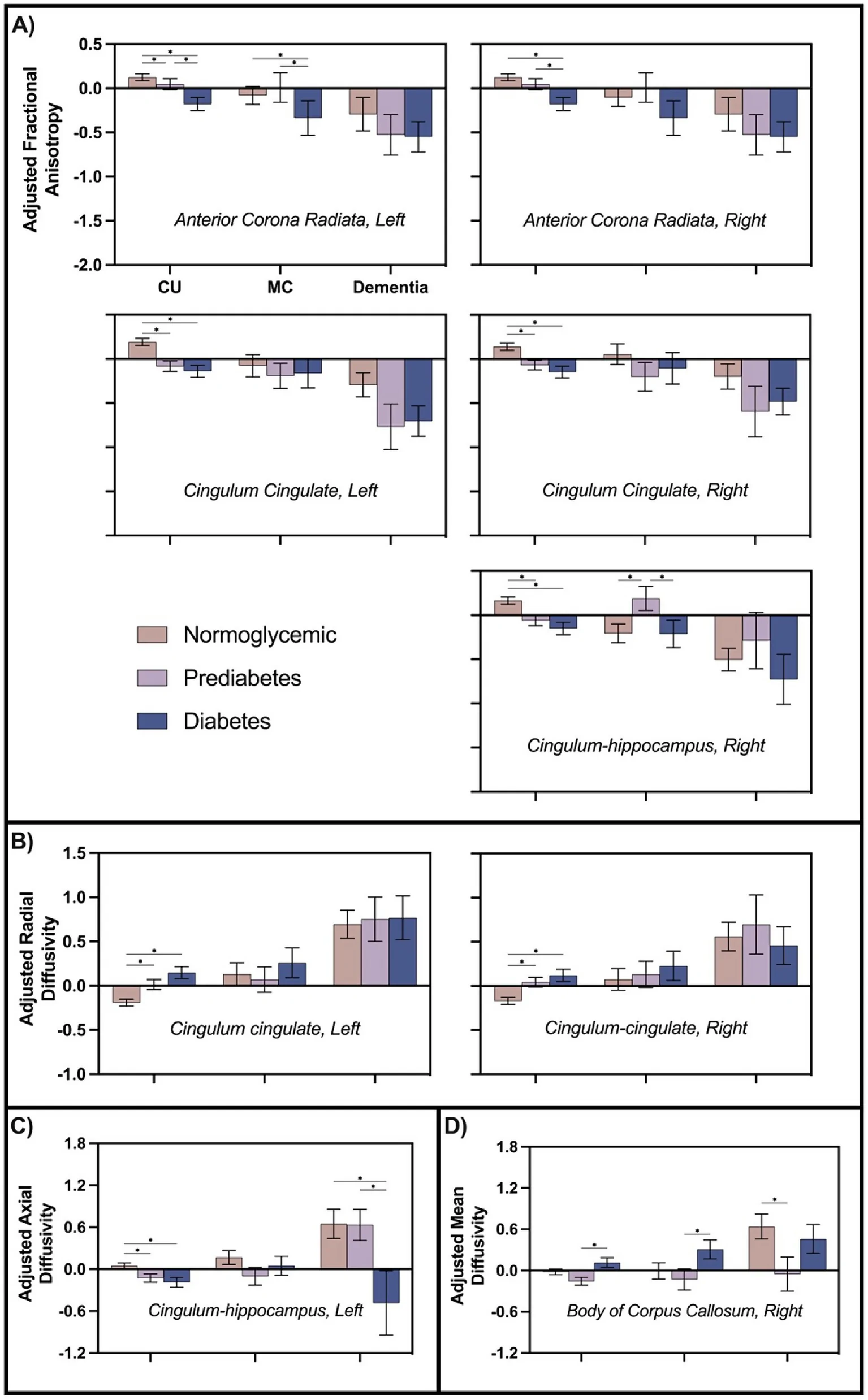
Cortical thickness and hippocampal volume measures across cognitive diagnostic and diabetes risk groupings. Graphs depicting means and standard errors of the mean for each cognitive diagnosis and diabetes grouping (determined by HbA1c). The *y*-axis represents covariate-adjusted (age, sex, and total intracranial volume) cortical thickness (or in the case of hippocampal regions, volume). All regions depicted in this figure showed an overall effect of cognition in the corresponding MANCOVA analysis (extensive results can be found in Supplementary Table 1). Pairwise differences are shown (*, Tukey’s adjusted *p*-value < 0.05). Pairwise comparisons were performed only between diabetes groupings within each cognitive diagnosis group. L, left; R, right; ROI, region-of-interest; Par, Parietal; STS, Superior Temporal Sulcus; Hippo, Hippocampus; CU, cognitively unimpaired; MCI, mild cognitive impairment.

### 3.2 White matter microstructure shows changes across cognition and glycemic status

We next examined DTI scalars for an effect of Cognition and HbA1c grouping. Detailed analysis of the DTI data in the HABS-HD cohort has been previously published (Hall et al., 2021). However, the composition of cohort used in this analysis differs slightly, based on exclusion process noted in Figure 1, and additionally evaluated shared effects of cognition and glycemic status. FA demonstrated shared effects of cognition and HbA1c grouping in bilateral corona radiata, bilateral cingulum-cingulate, and the right cingulum-hippocampus. These univariate tests are listed in Table 2. AxD showed shared effects only for left cingulum-hippocampus (Cognition, *F* = 8.018, *p* < 0.001; HbA1c, *F* = 8.564, *p* < 0.001). MD showed shared effects only for the body of the corpus callosum (Cognition, *F* = 7.43, *p* < 0.001; HbA1c, *F* = 7.155, *p* < 0.001). RD showed shared effects left cingulum-cingulate (Cognition, *F* = 25.409, *p* < 0.001; HbA1c, *F* = 9.094, *p* < 0.001) and right cingulum-cingulate (Cognition, *F* = 15.919, *p* < 0.001; HbA1c, *F* = 7.611, *p* < 0.001).

**Table 2:** MANCOVA results of DTI scalar analyses.

DTI Scalar summary statistic (*ROI*)	Cognition		Hba1c group	
	*F*	*p*-value	*F*	p-value
Fractional anisotropy				
Anterior corona radiata left	14.361	<0.001	12.833	<0.001
Cingulum cingulate gyrus left	12.81	<0.001	13.121	<0.001
Anterior corona radiata right	8.628	<0.001	7.774	<0.001
Cingulum cingulate gyrus right	5.619	0.002	9.921	<0.001
Cingulum hippocampus right	11.243	<0.001	6.828	0.001
Axial diffusivity				
Cingulum hippocampus left	8.018	<0.001	8.564	<0.001
Mean diffusivity				
Body of corpus callosum left	7.43	<0.001	7.155	<0.001
Radial diffusivity				
Cingulum cingulate gyrus left	25.409	<0.001	9.094	<0.001
Cingulum cingulate gyrus right	15.919	<0.001	7.611	<0.001

Table showing the results of the DTI scalar analyses. Each scalar was tested across a total of 32 different tracts, necessitating Sidak-adjustment across this number of comparisons. This table includes only the tracts which displayed significant effects for both cognitive diagnosis and diabetes grouping after this adjustment. All other findings are shown in detail in Supplementary Tables 2–5. Covariates included age, sex, and total intracranial volume. Scalars included fractional anisotropy, axial diffusivity, mean diffusivity, and radial diffusivity.

All tracts demonstrated associations with both factors showed significant Sidak-corrected pairwise comparisons between the CU, MCI, and dementia groups. Pairwise comparisons between the CU and MCI were significant for the FA of left anterior corona radiata, FA and RD of left cingulum-cingulate, FA of right cingulum-hippocampus, MD of the left body of the corpus callosum, and RD of left cingulum-cingulate. Pairwise comparisons between the MCI and group with dementia were significant for FA of left anterior corona radiata, FA and RD of left cingulum-cingulate, and RD of right Cingulum-cingulate. Thus, the scalar-tract pairings which show a clear, step-wise decreased between condition were FA of the anterior corona radiata (CU > MCI, *p* = 0.007; MCI > dementia, *p* < 0.001), FA of the cingulum-cingulate (CU > MCI, *p* = 0.042; MCI > dementia, *p* < 0.001), and RD of the cingulum-cingulate, left (CU < MCI, *p* = 0.013; MCI < dementia, *p* < 0.001) and right (CU < MCI, *p* = 0.046; MCI < dementia, *p* = 0.003).

All tracts which were tested significant for effects of both Cognition and HbA1c grouping were visualized for covariate-adjusted (age, sex, and intracranial volume) DTI scalar magnitude in Figure 4. Sidak-adjusted multiple comparisons between HbA1c groupings *within each diagnostic* category are shown. Notably, FA of the anterior corona radiata exhibited a stepwise decrease across HbA1c groupings within the cognitively unimpaired cohort (Normoglycemic > prediabetes, *p* = 0.047; prediabetes > diabetes, *p* = 0. 027).

**Figure 4:**
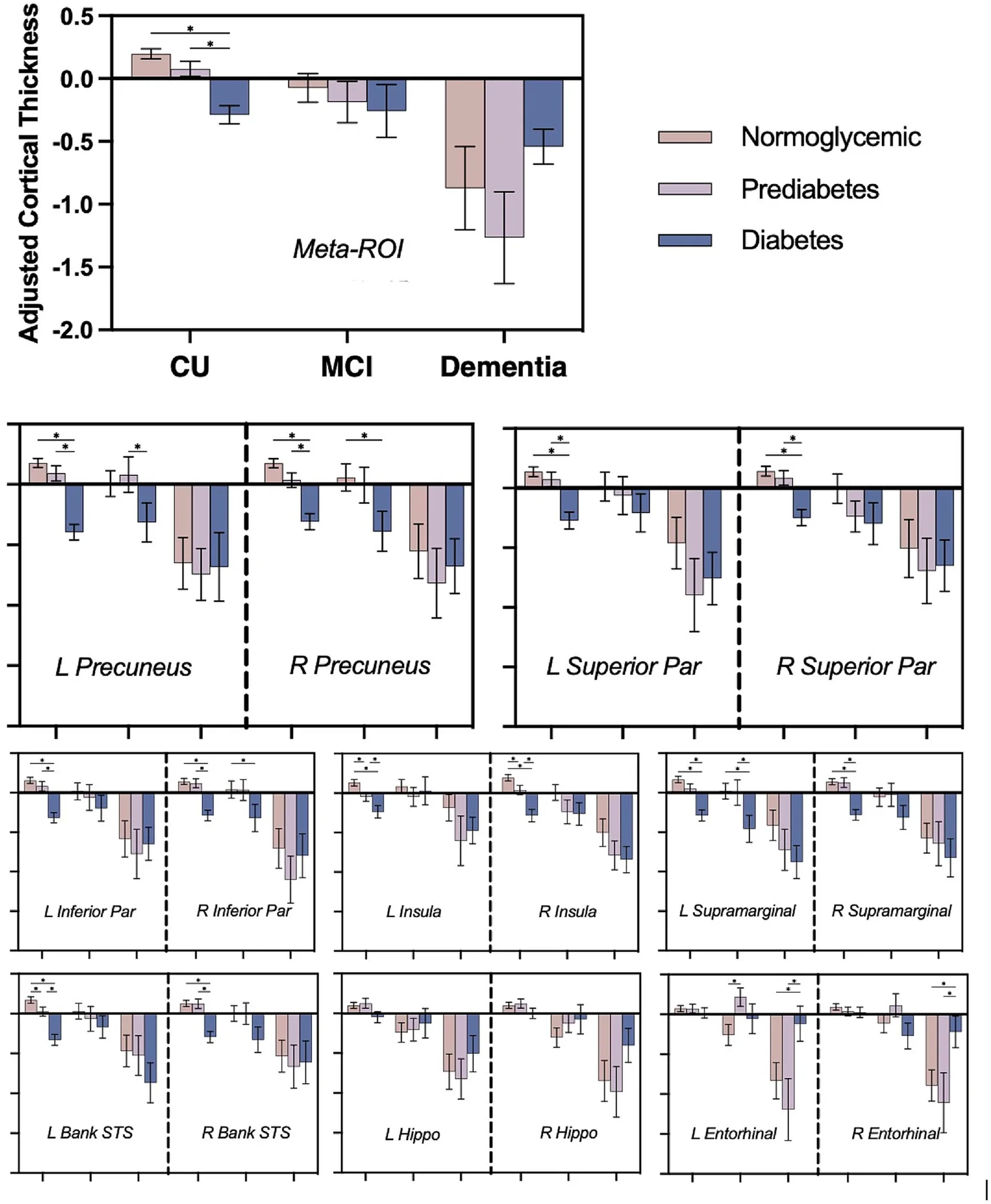
DTI results across tracts and scalars significant for cognitive diagnosis and hyperglycemia effects. Scalar means (error bars depict standard error of the mean) for tracts showing significant multiple comparisons-adjusted cognition and diabetes effects in the ANCOVA analyses. The *y*-axis represents covariate-adjusted (age, sex, and total intracranial volume) scalars. Pairwise differences are shown (*, Tukey’s adjusted *p*-value < 0.05). Pairwise comparisons were performed only between diabetes groupings within each cognitive diagnosis group.

### 3.3 DMN functional connectivity is selectively sensitive to cardiometabolic risk

We next examined functional connectivity within 6 DMN subnetworks for effects of Cognition and HbA1c grouping. Our findings are summarized in Table 3. Only two subnetworks demonstrated an effect of cognition: posterior DMN (pDMN, *F* = 7.762, *p* < 0.001) and dorsal DMN (dDMN, *F* = 3.357, *p* = 0.035). Pairwise comparisons revealed the pDMN demonstrated a decrease between CU vs. MCI (*p* = 0.003) and CU vs. group with dementia (*p* = 0.001), but not between the MCI and group with dementia (*p* = 0.259). In contrast, the tDMN did not show a significant pairwise decrease between unimpaired and MCI (*p* = 0.535), but did show a decrease between unimpaired vs. group with dementia (*p* = 0.012) and MCI vs. group with dementia (*p* = 0.010).

**Table 3:** MANCOVA results of DMN functional connectivity analyses.

DMN summary statistic	Cognition		Hba1c group	
	*F*	*p*-value	*F*	*p*-value
Posterior DMN	**7.762**	**<0.001**	**3.802**	**0.023**
Ventral DMN	0.204	0.816	0.007	0.993
Anterodorsal DMN	0.587	0.556	0.372	0.69
Anteroventral	0.878	0.416	2.938	0.053
Dorsal DMN	**3.357**	**0.035**	0.528	0.59
Temporal DMN	2.082	0.125	1.894	0.151

Table showing the results of the functional connectivity MANCOVA analyses in which effects of cognitive diagnosis and diabetes grouping was tested. Bolded *F*-statistics and *p*-values represent those which were found to be significant after Sidak-adjustment across the six comparisons. Covariates included age, sex, total intracranial volume, and absolute motion during functional scan.

Only the pDMN demonstrated an effect of both Cognition and HbA1c grouping (*F* = 3.802, *p* = 0.023). Pairwise comparisons showed that the normoglycemic group differed significantly from the diabetes group (*p* = 0.029), but did not show differences between normoglycemia and prediabetes or prediabetes and diabetes. We then examined MetS-Z score against the functional connectivity summary metrics. These findings are summarized in Table 4 for the cognitively unimpaired cohort. The pDMN was shown to decrease linearly with increasing MetS-Z score (*t* = −3.018, *p* = 0.003). Interestingly, the dDMN appeared to positively associate with MetS-Z (*t* = 2.737, *p* = 0.006) as did two other subnetworks, the anterior dorsal DMN (adDMN, *t* = 3.415, *p* < 0.001) and the anterior ventral DMN (avDMN, *p* < 0.001). Models testing the effect of MetS-Z score on functional connectivity of DMN subnetworks in the MCI and dementia cohorts showed no significant associations.

**Table 4:** MetS-Z score effect on DMN functional connectivity in the cognitively unimpaired cohort.

DMN summary statistic cognitively health only	MetS-*Z*	
	Beta	*p*-value
Posterior DMN	**-3.018**	**0.003**
Ventral DMN	1.081	0.28
Anterodorsal DMN	**3.415**	**<0.001**
Anteroventral DMN	**3.735**	**<0.001**
Dorsal DMN	**2.737**	**0.006**
Temporal DMN	2.039	0.042

Table showing the results of the MANCOVA analyses in which the effect of metabolic Z-score (MetS-Z) on functional connectivity in the cognitively unimpaired was tested. Bolded betas and *p*-values represent those which were found to be significant after Sidak-adjustment across the six comparisons. Covariates included age, sex, total intracranial volume, and absolute motion during functional scan.

## 4 Discussion

This study provides a comprehensive examination of structural, microstructural, and functional neuroimaging biomarkers of Alzheimer’s disease (AD) across cognitive diagnoses and metabolic risk strata in a large, ethnically diverse cohort. Building on prior work from our group and others, these findings reinforce the concept that metabolic dysfunction meaningfully influences brain health and may contribute to AD risk well before the onset of clinical cognitive impairment.

Our group has previously demonstrated that impaired glycemic regulation is associated with accelerated cognitive decline and structural brain changes. Using ADNI data, we showed worse cognitive trajectories in individuals with impaired fasting glucose, consistent with broader evidence linking metabolic dysfunction to cognitive decline (Morris et al., 2014). This is consistent with other work demonstrating accelerations in cognitive decline in patients with metabolic dysfunction (Luchsinger et al., 2007; Arvanitakis et al., 2004). In a group of 20 individuals with AD, 22 individuals with Parkinson’s Disease, and 21 healthy controls, insulin resistance tracked negatively with brain volume, with stronger metabolic derangement appearing in the AD group (9). We have also linked impaired glycemia and insulin resistance to changes in brain structure. In early stage AD (CDR of 0.5 and 1), we found insulin and glucose AUC associated with decreases in hippocampal and global brain volume (Burns et al., 2007). The present study extends this work by integrating multimodal neuroimaging with both categorical and continuous measures of metabolic risk, with a specific focus on Default Mode Network (DMN) organization.Structural and diffusion imaging analyses demonstrated that metabolic dysfunction is associated with compromised brain integrity even in cognitively unimpaired individuals. Cortical thickness reductions were observed in regions classically vulnerable to AD, and white matter microstructure—particularly within DMN-associated tracts—showed stepwise degradation across glycemic categories. Notably, individuals with prediabetes frequently exhibited intermediate phenotypes, suggesting that microstructural brain changes may emerge prior to overt diabetes. Such changes may reflect microstructural integrity, white matter health, and myelination, which may inform a link between cardiometabolic health and neurodegeneration across multiple dementia etiologies (O'Donnell and Westin, 2011; Kantarci et al., 2017). While these effects were most robust in cognitively unimpaired participants, the absence of significant metabolic effects within the dementia group may reflect reduced statistical power or the predominance of downstream neurodegenerative processes at later disease stages. Additionally, reliance on clinical diagnoses represents a limitation that future work incorporating plasma biomarkers may help address.

Functional connectivity analyses revealed a nuanced, network-specific relationship between metabolic risk and DMN organization. Functional connectivity has demonstrated key changes in the networks of AD patients (Seeley et al., 2009; Buckner et al., 2009; Misiura et al., 2020; Corriveau-Lecavalier et al., 2023), which may in fact occur years before clinical decline begins to become evident. However, the directionality of effect of traditional AD neuropathology (amyloid-β) on functional connectivity in cognitively unimpaired adults remains mixed, with reporting of both increases and decreases in connectivity (Buckner et al., 2009; Ingala et al., 2021; Sperling et al., 2009; Tahmi et al., 2020; Buckner et al., 2005). A prior meta-analysis of 19 independent fMRI studies found type 2 diabetes diagnosis was independently associated with decreased connectivity in the DMN (Macpherson et al., 2017). Type 2 diabetes also predicted reduced connectivity between the posterior cingulate cortex (PCC) and hippocampus, a feature shared by AD. In those with type 2 diabetes, reduced connectivity of regions of the anterior and posterior DMN was linked to poorer performance on neuropsychological tasks. This may indicate that these functional connectivity changes reflect meaningful derangements of underlying neural processes (Nickerson et al., 2017; Hillary and Grafman, 2017).

In the present study, we demonstrate decreases in connectivity of the posterior DMN (pDMN) associated with both cognitive impairment and hyperglycemia. While hyperglycemia only was not associated with changes in connectivity of any other DMN subnetworks, our evaluation of MetS-Z score showed that a more comprehensive measure of cardiometabolic risk was associated with increased connectivity in dDMN, avDMN, and dDMN in cognitively unimpaired individuals. These divergent patterns suggest that metabolic dysfunction may exert heterogeneous effects across DMN subnetworks, with early vulnerability of posterior hubs accompanied by compensatory or maladaptive hyperconnectivity in other regions that is detectable only when broader systemic cardiometabolic burden is considered.

Our findings add to existing work supporting the value of continuous metabolic risk indices for detecting subtle brain–metabolism relationships and highlights cardiometabolic dysfunction as a potentially modifiable contributor to early neural vulnerability. In a longitudinal study of individuals with a family history of AD, vascular risk factors including higher total cholesterol, higher low-density lipoprotein (LDL), and higher diastolic blood pressure (DBP) were associated with functional network changes when amyloid-β and tau were not (Köbe et al., 2020). The observation of increased connectivity in select DMN subnetworks aligns with prior reports of hyperconnectivity in individuals at risk for AD (Hillary and Grafman, 2017; Green et al., 2023; Schultz et al., 2017) and may reflect functional reorganization of large-scale networks in response to metabolic or vascular stress (Jones et al., 2016). Future efforts should prioritize the use of integrative, continuous measures of cardiometabolic risk to better delineate how cumulative vascular and metabolic burden shapes early functional network reorganization preceding overt neurodegeneration.

Several limitations should be considered when interpreting these findings. First, the cross-sectional design precludes inference regarding temporal or causal relationships between cardiometabolic dysfunction and changes in brain structure or functional connectivity. The cross-sectional design precludes causal inference, and unexamined factors such as medication use or specific vascular comorbidities uncaptured by MetS-Z score may contribute to observed associations. In addition, while the use of MetS-Z is helpful in evaluating continuous associations between risk severity and our outcomes of interest, this metric does not readily translate to traditional MetS categories (DeBoer et al., 2017). This means that clinical interpretability is limited. Moreover, interpretation of cardiometabolic risk effects related to the examined neuroimaging measures was also limited within the MCI and dementia groups due to large variability in the outcomes. This could be driven by the fact that, despite the size of the overall HABS-HD cohort, the number of participants with clinically diagnosed dementia was relatively small, which may have limited statistical power to detect metabolic effects within this group and contributed to greater variability, especially in functional connectivity measures. Additionally, clinical diagnoses of MCI and dementia are inherently heterogeneous and may encompass individuals with differing underlying neuropathologies, potentially obscuring more specific associations. Future studies integrating longitudinal neuroimaging, plasma and neuroimaging biomarkers of AD pathology, vascular imaging, and more granular metabolic and treatment data will be critical to disentangling these interrelated factors and clarifying mechanisms linking cardiometabolic dysfunction to brain network organization. Finally, the continuous MetS-Z measure was evaluated in relation to functional connectivity. Although glycemic status was evaluated in regards to structure, future work is needed to capture the relationship between broader MetS-Z measures and structural outcomes.

A major strength of this study is the inclusion of a large and ethnically diverse cohort, addressing a persistent limitation in Alzheimer’s disease and clinical neuroimaging research. Inadequate recruitment of underrepresented populations has constrained the generalizability of prior work, despite well-established evidence that ethnicity influences both dementia risk and cardiometabolic burden through differences in healthcare access, lifestyle factors, and exposure to race-related stressors (Williams, 2018). Prior research has demonstrated that Mexican Americans present with dementia at younger ages, and often with a higher burden of cognitive and behavioral change (O'Bryant et al., 2021; O’Bryant et al., 2007). Moreover, this population has been shown to express a distinct “metabolic phenotype” of AD biomarker profiles and to experience higher rates of metabolic dysfunction (O'Bryant et al., 2013), and suffer from a higher rate of metabolic dysfunction (Aguayo-Mazzucato et al., 2019; Filshtein et al., 2019). By leveraging the HABS-HD cohort, the present study represents one of the first efforts to characterize multimodal neuroimaging biomarkers—particularly functional connectivity within the Default Mode Network—across cognitive diagnoses and metabolic risk strata in a large, diverse sample of older adults. This inclusive design enhances the translational relevance of the findings and supports the need for future AD research to explicitly incorporate metabolic and vascular risk within diverse populations. It should be acknowledged that the field is moving towards biomarker definitions of AD, although there remains some uncertainty about these applications in underrepresented populations (Leuzy et al., 2025; Schindler et al., 2022). However, because we did not include amyloid or tau biomarkers, our findings should not be interpreted as describing biomarker defined AD. We demonstrate associations between cardiometabolic burden and neuroimaging phenotypes in clinically informed diagnosis groups only. Future work is needed to confirm these relationships in biomarker confirmed AD. In addition, while the MetS-Z score accounts for the severity of each component, to our knowledge clinically meaningful MetS-Z score cutpoints have not been validated.

In conclusion, this study demonstrates that cardiometabolic dysfunction is associated with early, network-specific alterations in brain structure and functional connectivity across the cognitive spectrum. Using multimodal neuroimaging in a large and diverse cohort, we show that metabolic risk—particularly when captured using integrative, continuous indices—relates to vulnerability of posterior Default Mode Network hubs even in cognitively unimpaired individuals, while other DMN subnetworks exhibit patterns consistent with functional reorganization. Though this study’s cross-sectional design is insufficient to establish causal direction, the findings would be expected in the case that cardiometabolic burden represents an important and potentially modifiable contributor to neural changes that precedes overt neurodegeneration. Continued longitudinal and mechanistic studies integrating metabolic, vascular, and molecular biomarkers will be critical to clarifying causal pathways and informing strategies aimed at reducing dementia risk through targeted metabolic health interventions.

## Data Availability

The datasets presented in this study can be found in online repositories. The names of the repository/repositories and accession number(s) can be found at: HABS-HD, https://apps.unthsc.edu/itr/researchers.
