# Supplementary material for: Metabolic dysfunction associates with neuroimaging measures of brain structure and connectivity
Source: Front Neurosci. 2026 Sep 25;20:1889255. doi: 10.3389/fnins.2026.1889255 (PMC13649970; doi:10.3389/fnins.2026.1889255)
Supplement: Supplementary file 1 [file Table_1.docx]

Online Supplemental Data

**Supplementary Table 1. All cortical thickness MANCOVA results**

|  | *Cognition* | | *Hba1c group* | |
| --- | --- | --- | --- | --- |
| *Region* | *F* | *p-value* | *F* | *p-value* |
| Left hippocampus | **13.632** | **<0.001** | 0.96 | 0.383 |
| Right hippocampus | **15.312** | **<0.001** | 0.292 | 0.747 |
| **Left bank of STS** | **12.381** | **<0.001** | **18.053** | **<0.001** |
| **Right bank of STS** | **10.102** | **<0.001** | **11.121** | **<0.001** |
| Left entorhinal | **9.955** | **<0.001** | 0.412 | 0.662 |
| Right entorhinal | **14.229** | **<0.001** | 0.13 | 0.878 |
| **Left inferior parietal** | **10.139** | **<0.001** | **14.008** | **<0.001** |
| **Right inferior parietal** | **12.627** | **<0.001** | **9.664** | **<0.001** |
| Left posterior cingulate | 1.156 | 0.315 | 3.668 | 0.026 |
| Right posterior cingulate | 0.665 | 0.515 | 2.743 | 0.065 |
| **Left precuneus** | **11.195** | **<0.001** | **20.623** | **<0.001** |
| **Right precuneus** | **9.533** | **<0.001** | **13.721** | **<0.001** |
| Left superior frontal | 2.256 | 0.105 | 4.436 | 0.012 |
| Right superior frontal | 1.916 | 0.148 | 4.892 | 0.008 |
| **Left superior parietal** | **10.021** | **<0.001** | **10.623** | **<0.001** |
| **Right superior parietal** | **8.061** | **<0.001** | **7.699** | **<0.001** |
| **Left superior temporal** | **10.038** | **<0.001** | **17.191** | **<0.001** |
| **Right superior temporal** | **8.901** | **<0.001** | **17.066** | **<0.001** |
| **Left supramarginal** | **9.484** | **<0.001** | **14.782** | **<0.001** |
| **Right supramarginal** | **10.626** | **<0.001** | **10.559** | **<0.001** |
| Left insula | 0.655 | 0.52 | 5.354 | 0.005 |
| **Right insula** | **14.885** | **<0.001** | **15.923** | **<0.001** |
| **Cortical Thickness Meta-ROI** | **19.246** | **<0.001** | **9.515** | **<0.001** |

**Supplementary Table 1. All cortical thickness MANCOVA results.** Table showing the extensive results of MANCOVA analyses of the effects of cognitive diagnosis and diabetic grouping on cortical thickness of different regions. Bolded F-statistics and p-values represent those which were found to be significant after Sidak-adjustment across the multiple comparisons made. Results which were significant for both an effect of cognition and diabetic grouping went on to be included in Figure 3. Covariates included age, gender, and total intracranial volume. Abbreviations: STS, superior temporal sulcus; ROI, region-of-interest.
