# Supplementary material for: Metabolic dysfunction associates with neuroimaging measures of brain structure and connectivity
Source: Front Neurosci. 2026 Sep 25;20:1889255. doi: 10.3389/fnins.2026.1889255 (PMC13649970; doi:10.3389/fnins.2026.1889255)
Supplement: Supplementary file 2 [file Table_2.docx]

**Supplementary Table 2. All tracts differing in FA across cognition**

| *Fractional Anisotropy* | *Cognition* | | *Hba1c group* | |
| --- | --- | --- | --- | --- |
| *Estimates significant for only Diagnosis effect* | *F* | *p-value* | *F* | *p-value* |
| Posterior thalamic radiation left | 7.961 | <0.001 | 0.274 | 0.76 |
| Cingulum hippocampus left | 15.013 | <0.001 | 4.426 | 0.012 |
| Inferior fronto occipital fasciculus left | 7.243 | <0.001 | 4.758 | 0.009 |
| Body of corpus callosum left | 9.776 | <0.001 | 4.974 | 0.007 |
| Genu of corpus callosum left | 14.187 | <0.001 | 0.924 | 0.397 |
| Splenium of corpus callosum left | 15.587 | <0.001 | 0.002 | 0.998 |
| Posterior thalamic radiation right | 12.841 | <0.001 | 1.196 | 0.303 |
| Inferior fronto occipital fasciculus right | 7.026 | <0.001 | 6.672 | 0.001 |
| Genu of corpus callosum right | 13.683 | <0.001 | 2.21 | 0.11 |
| Body of corpus callosum right | 9.805 | <0.001 | 3.119 | 0.045 |
| Splenium of corpus callosum right | 11.696 | <0.001 | 0.677 | 0.508 |

**Supplementary Table 2. All tracts differing in FA across cognition.** Table showing the results of MANCOVA analyses of the effects of cognitive diagnosis and diabetic grouping on fractional anisotropy (FA) of different tracts. An extensive list of all tracts tested is included in the Methods section. Each scalar was tested across a total of 32 different tracts, necessitating Sidak-adjustment across this number of comparisons. Only regions which demonstrated an effect of at least cognition after multiple comparisons-adjustment are included in this table. Bolded F-statistics and p-values represent those which were found to be significant after Sidak-adjustment across the multiple comparisons made. Results which were significant for both an effect of cognition and diabetic grouping went on to be included in Table 2 and Figure 4. Covariates included age, gender, and total intracranial volume.
