# Supplementary material for: Metabolic dysfunction associates with neuroimaging measures of brain structure and connectivity
Source: Front Neurosci. 2026 Sep 25;20:1889255. doi: 10.3389/fnins.2026.1889255 (PMC13649970; doi:10.3389/fnins.2026.1889255)
Supplement: Supplementary file 5 [file Table_5.docx]

**Supplementary Table 5. All tracts differing in RD across cognition**

| *Radial Diffusivity* | *Cognition* | | *Hba1c group* | |
| --- | --- | --- | --- | --- |
| *Estimates significant for only Diagnosis effect* | *F* | *p-value* | *F* | *p-value* |
| Anterior limb of internal capsule left | 9.309 | <0.001 | 2.994 | 0.05 |
| Posterior thalamic radiation left | 20.58 | <0.001 | 1.307 | 0.271 |
| Anterior corona radiata left | 22.844 | <0.001 | 3.49 | 0.031 |
| Cingulum hippocampus left | 37.667 | <0.001 | 0.284 | 0.753 |
| Superior longitudinal fasciculus left | 10.804 | <0.001 | 0.369 | 0.692 |
| Superior fronto occipital fasciculus left | 16.523 | <0.001 | 2.546 | 0.079 |
| Inferior fronto occipital fasciculus left | 23.285 | <0.001 | 5.679 | 0.004 |
| Uncinate fasciculus left | 16.299 | <0.001 | 1.299 | 0.273 |
| Genu of corpus callosum left | 17.675 | <0.001 | 1.402 | 0.246 |
| Body of corpus callosum left | 8.561 | <0.001 | 6.36 | 0.003 |
| Splenium of corpus callosum left | 24.993 | <0.001 | 0.987 | 0.373 |
| Anterior limb of internal capsule right | 12.269 | <0.001 | 0.055 | 0.946 |
| Posterior thalamic radiation | 22.166 | <0.001 | 0.934 | 0.393 |
| Anterior corona radiata right | 17.094 | <0.001 | 1.6 | 0.202 |
| Cingulum hippocampus right | 42.075 | <0.001 | 4.018 | 0.018 |
| Superior longitudinal fasciculus right | 6.863 | 0.001 | 1.175 | 0.309 |
| Inferior fronto occipital fasciculus right | 18.281 | <0.001 | 2.785 | 0.062 |
| Uncinate fasciculus right | 13.65 | <0.001 | 0.112 | 0.894 |
| Genu of corpus callosum right | 19.333 | <0.001 | 1.18 | 0.307 |
| Body of corpus callosum right | 10.429 | <0.001 | 2.599 | 0.075 |
| Splenium of corpus callosum right | 24.744 | <0.001 | 0.02 | 0.98 |

**Supplementary Table 5. All tracts differing in RD across cognition.** Table showing the results of MANCOVA analyses of the effects of cognitive diagnosis and diabetic grouping on radial diffusivity (RD) of different tracts. An extensive list of all tracts tested is included in the Methods section. Each scalar was tested across a total of 32 different tracts, necessitating Sidak-adjustment across this number of comparisons. Only regions which demonstrated an effect of at least cognition after multiple comparisons-adjustment are included in this table. Bolded F-statistics and p-values represent those which were found to be significant after Sidak-adjustment across the multiple comparisons made. Results which were significant for both an effect of cognition and diabetic grouping went on to be included in Table 2 and Figure 4. Covariates included age, gender, and total intracranial volume.
